# Growth effects of exclusive breastfeeding promotion by peer counsellors in sub-Saharan Africa: the cluster-randomised PROMISE EBF trial

**DOI:** 10.1186/1471-2458-14-633

**Published:** 2014-06-21

**Authors:** Ingunn Marie Stadskleiv Engebretsen, Debra Jackson, Lars Thore Fadnes, Victoria Nankabirwa, Abdoulaye Hama Diallo, Tanya Doherty, Carl Lombard, Sonja Swanvelder, Jolly Nankunda, Vundli Ramokolo, David Sanders, Henry Wamani, Nicolas Meda, James K Tumwine, Eva-Charlotte Ekström, Philippe Van de Perre, Chipepo Kankasa, Halvor Sommerfelt, Thorkild Tylleskär

**Affiliations:** 1 Centre for International Health, Department of Global Public Health and Primary Care, University of Bergen, PO Box 7804, 5020 Bergen, Norway; 2 School of Public Health, University of Western Cape, P Bag X17, Bellville 7535, South Africa; 3 Department of Clinical Dentistry, University of Bergen, PO Box 7804, 5020 Bergen, Norway; 4 Department of Epidemiology, Mailman School of Public Health, Columbia University, New York, NY 10032, USA; 5 School of Public Health, Makerere University, Kampala, Uganda; 6 Centre MURAZ, Ministry of Health, PO Box 390, Bobo-Dioulasso, Burkina Faso; 7 Health Systems Research Unit, Medical Research Council, 7505 Cape Town, South Africa; 8 Biostatistics Unit, Medical Research Council, 7505 Cape Town, South Africa; 9 Department of Paediatrics and Child Health, College of Health Sciences, Makerere University, Kampala, Uganda; 10 Department of Women’s and Children’s Health, Uppsala University, Uppsala, Sweden; 11 INSERM U1058, Montpellier, France; 12 Université de Montpellier I, Montpellier, France; 13 Centre Hospitalier Universitaire Montpellier, Département de Bactériologie-Virologie, Montpellier, France; 14 Department of Paediatrics and Child Health, School of Medicine, University of Zambia, Lusaka, Zambia; 15 Department of International Public Health, Norwegian Institute of Public Health, N-0403 Oslo, Norway

**Keywords:** Exclusive breastfeeding promotion, Peer counselling, Child growth, Anthropometry, Stunting, Wasting, Underweight, Undernutrition, Community randomised trial

## Abstract

**Background:**

In this multi-country cluster-randomized behavioural intervention trial promoting exclusive breastfeeding (EBF) in Africa, we compared growth of infants up to 6 months of age living in communities where peer counsellors promoted EBF with growth in those infants living in control communities.

**Methods:**

A total of 82 clusters in Burkina Faso, Uganda and South Africa were randomised to either the intervention or the control arm. Feeding data and anthropometric measurements were collected at visits scheduled 3, 6, 12 and 24 weeks post-partum. We calculated weight-for-length (WLZ), length-for-age (LAZ) and weight-for-age (WAZ) z-scores. Country specific adjusted Least Squares Means with 95% confidence intervals (CI) based on a longitudinal analysis are reported. Prevalence ratios (PR) for the association between peer counselling for EBF and wasting (WLZ < −2), stunting (LAZ < −2) and underweight (WAZ < −2) were calculated at each data collection point.

**Results:**

The study included a total of 2,579 children. Adjusting for socio-economic status, the mean WLZ at 24 weeks were in Burkina Faso −0.20 (95% CI −0.39 to −0.01) and in Uganda −0.23 (95% CI −0.43 to −0.03) lower in the intervention than in the control arm. In South Africa the mean WLZ at 24 weeks was 0.23 (95% CI 0.03 to 0.43) greater in the intervention than in the control arm. Differences in LAZ between the study arms were small and not statistically significant. In Uganda, infants in the intervention arm were more likely to be wasted compared to those in the control arm at 24 weeks (PR 2.36; 95% CI 1.11 to 5.00). Differences in wasting in South Africa and Burkina Faso and stunting and underweight in all three countries were small and not significantly different.

**Conclusions:**

There were small differences in mean anthropometric indicators between the intervention and control arms in the study, but in Uganda and Burkina Faso, a tendency to slightly lower ponderal growth (weight-for-length z-scores) was found in the intervention arms.

**Trial registration number:**

ClinicalTrials.gov: NCT00397150

## Background

The World Health Organization (WHO) has since 2001 recommended exclusive breastfeeding (EBF) for the first six months of life [1]. It is estimated that universal coverage with general nutritional interventions including exclusive breastfeeding promotion could prevent 8% of child deaths under the age of 36 months and 10-15% of stunting [2]. The systematic reviews by Kramer *et al*. reported that EBF for 6 months compared to EBF for 3 to 4 months resulted in lower diarrheal morbidity, prolonged lactational amenorrhoea and no clear infant growth deficit among infants in either low- or high income countries [3,4]. However, few African studies were included in that review and the authors stated that “larger sample sizes would be required to rule out modest increases in the risk of undernutrition” with longer duration than 3–4 months of EBF [3,5].

A recent systematic review reported that peer support for breastfeeding decreased the risk of non-exclusive breastfeeding at last study-follow-up by 37% in low- and middle income countries compared to only 10% in high income countries [4]. Even if breastfeeding promotion can substantially increase the proportion of infants that are breastfed [6,7], its effect on growth is less clear. It is assumed that breastfed infants are healthier than non-breastfed infants [2,8,9]. This is expected to result from reduced incidence and severity of communicable diseases, including diarrhoea [6,8] and improved feeding during illness [6,9,10]. Other expected benefits are lower risk of autoimmune diseases [11] and potential long term beneficial effects including on cognition [12-15]. In addition, formula fed infants may receive excess energy, which could lead to overweight in childhood, and potentially later in life [10,16,17].

This cluster-randomised trial estimated the effect of community-based promotion of EBF by peer counsellors on the prevalence of EBF and diarrhea [18]. In order to address whether EBF promotion could impact infant growth, this paper reports on growth patterns including ponderal growth (expressed as weight-for-length z-scores) and linear growth (length-for-age z-scores) as well as weight-for-age z-scores up to 6 months of age in children participating in the PROMISE EBF trial in Burkina Faso, Uganda and South Africa. Our research question was whether there was any significant difference in growth patterns between the intervention and control clusters by 24 weeks of age.

## Methods

The effect of peer counselling on EBF and diarrhoea prevalence and the methods used in the PROMISE EBF trial are described elsewhere [18]. Briefly, the study was conducted in three countries: Burkina Faso, Uganda and South Africa. In Burkina Faso, the study was conducted in Banfora, a south-western rural area dominated by subsistence farming. In Uganda, the sites comprised rural Bungokho in Mbale District where both subsistence farming and petty trading are common, and urban Mbale Municipality characterised by informal settlements and small industries. There were three geographically separate sites in South Africa: 1) Paarl, a commercial farming area in the Western Cape Province, 2) peri-urban Umlazi, and 3) rural Rietvlei in KwaZulu-Natal. Infant mortality rates (IMRs) at the time of the study were 92/1,000 in Burkina Faso and 85/1,000 in Uganda. In South Africa, the IMRs were 40/1,000 in Paarl, 60/1,000 in Umlazi and 99/1,000 in Rietvlei [19].

Within each country and site, clusters were randomised 1:1 to either the intervention (provision of EBF peer-counselling) or the control arm (where EBF was not promoted by our research team) [18]. This was also the case in South Africa, and in addition a separate team of peer supporters supported the families to obtain birth certificates and social welfare grants in the control clusters [20]. This was believed not to interfere with breastfeeding behaviour. Clusters were selected geographically to reduce contamination of the intervention.

The intervention consisted of EBF counselling by peer-counsellors that were from the local communities and trained in a one week course by the national research teams with a curriculum adapted from the WHO courses ‘*Breastfeeding Counselling: a Training Course*’ [21] and ‘*HIV & Infant Feeding Counselling: a Training Course*’ [22]. All mothers were offered at least five home visits, the first occurred in the third trimester of pregnancy. Qualitative descriptions of the intervention are given elsewhere [23-25].

**Figure 1:**
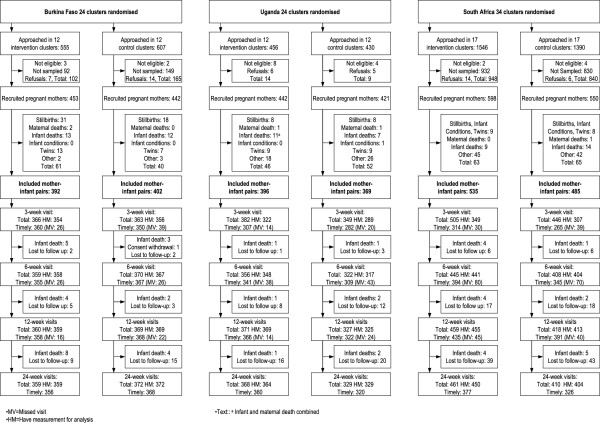
Promise EBF trial profile in Burkina Faso, Uganda and South Africa.

Sample size calculation was done for EBF and diarrhea prevalence [18]. Those intending to breastfeed and planning to stay in the selected cluster in the forthcoming year were included, further details on recruitment, inclusion and exclusion are given in the Additional file 1. The analysis included 2,579 singleton live children. The trial profile is given in Figure 1.

Data were collected between 2006 and 2008 using a recruitment interview late in pregnancy, and further interviews targeted at weeks 3, 6, 12 and 24 after birth. Structured questionnaires were developed and adapted from the literature [26,27] and informed by prior work in the participating countries [28-30]. The first recruitment interview focused mainly on socio-demographic and socio-economic characteristics. The follow-up contact points captured mother-reported feeding practices and infant illness, and the children’s length and weight were recorded. Interviews were regarded as ‘timely’ if they were done within the following time periods: 1.5–4.5 (3); 4.5–9 (6); 9–18 (12); and 18–28 (24) weeks. Timely measurements provided data for the undernutrition prevalence estimates. The trial profile reflects number of ‘timely’ out of ‘total’ interviews per scheduled visit (Figure 1). The trial profile also reflects the number of anthropometric measurements, all measurements went into the longitudinal modelling of the anthropometric data.

Length was measured to the nearest 0.1 cm using ‘Shorr Height-Length Measuring Board’ (Maryland, USA) provided as ‘Baby/infant/adult Length-height measuring system SET 2’ from UNICEF in Uganda, and ‘Seca 210 mobile measuring mats for babies and toddlers,’ with 0.5 cm precision, in Burkina Faso and TALC’s roller mat infantometer (Oxford, UK) in South Africa. Infant weight was recorded to the nearest 0.1 kg using the ‘Infant scale spring type, 25 kg, 100 g’ from UNICEF in Uganda, the ‘SECA 872 scale with mother-infant children’s’ function’ in Burkina Faso and ‘Mascott electronic bucket’ in South Africa. The measurement procedures followed standard WHO guidelines [31]. All data collectors underwent training in content, questionnaire techniques and measurements and were kept uninformed about cluster allocation. For the weight and length measurements, reproducibility and validity exercises were conducted. Re-training and evaluation were done at least semi-annually during the data collection period. The acceptable technical error of measurement (TEM) for a data collector [32] was a value less than two times that of the data collector supervisor, a clinical specialist.

### Definitions

A strict definition of exclusively breastfeeding (EBF) was applied. Infants were classified as EBF if they did not receive food or liquids other than breast milk except for medicines using a 24-hour and 7-day recall at 12 and 24 weeks of age [18]. WHO Child Growth Standards (2006) were used to estimate anthropometric status [33]: weight-for-length z-scores (WLZ), length-for-age z-scores (LAZ) and weight-for-age z-scores (WAZ). Children who had WLZ below −2 (WLZ < −2) were considered wasted, those with LAZ below −2 (LAZ < −2) stunted, and those with WAZ below −2 (WAZ < −2) underweight. Attribution of socio-economic status was based on within country ranking of groupings of different household commodities as variables in a multiple correspondence analysis, a technique which is suitable for categorical variables and similar to principal component analysis [34].

### Data handling and analysis

Descriptive statistics included means with 95% confidence intervals, medians and ranges for continuous variables and prevalence estimates with 95% confidence intervals for categorical variables. The data were analysed using STATA SE11.0 (Stata Corp LP, College Station, TX, USA) and SAS V9.2 (SAS Institute Inc., Cary, NC, USA).

Missed visits, the fact that some mothers did not consent to measurements at all visits, and the data cleaning process resulted in some missing anthropometric data. A detailed description of data cleaning and handling of missing anthropometric information is presented in the Additional file 1. An *inverse-probability weighted method* (IPW) was used instead of a plain *available-subject-analysis* to adjust for potential differences in follow-up between groups (Additional file 1).

Analysis was done by country and estimates took into account the design effect for having randomized *clusters* rather than individuals; for Uganda and South Africa we also adjusted for *site*. For the categorical anthropometric outcomes, generalised linear models (binomial family with a log link) were used to calculate prevalence ratios (PR). In the longitudinal analysis, the correlated nature of the data from the repeated measurements was taken into account by adjusting for repeated measurements in the same individual as well as the above-mentioned design effect resulting from the cluster randomization. A linear mixed effects regression model (PROC MIXED in SAS) was used in the longitudinal analysis of z-scores with cluster as a random effect and the repeated measurements in each child accounted for through a first order autocorrelation structure. Least Squares Means (LSM), which are analogous to estimated marginal means, are reported at 3, 6, 12 and 24 weeks, corresponding to the scheduled data collection visits. This paper presents the growth data in line with the trial design (intention-to-treat), and is not restricted to those mother-infant pairs who actually received per counselling and the frequency or duration of counselling is not considered in the models. Acknowledging that socio-economic status can affect growth [35], we also present data adjusted for socio-economic status. The study team planned to present the growth data by country acknowledging the large country differences in socio-economic status, feeding and health related factors [18].

### Ethical approval

Approval for the trial (ClinicalTrials.gov: NCT00397150) was obtained from the following bodies: 1) Burkina Faso: the Institutional Review board of Centre Muraz (No 013/2005/CE-CM) and from the Ministry of Health at national and regional level; 2) Uganda: Makerere University Faculty of Medicine Research and Ethics Committee, and the Uganda National Council for Science and Technology; 3) South Africa: Ethics Committee of the Medical Research Council South Africa; and 4) Norway: Regional Committees for Medical and Health Research Ethics (REK VEST), 8 Sept 2005, issue number 05/8197. Women provided verbal informed consent for participation in the peer-counselling programme, which was regarded as a service. Written informed consent for participation in the study was signed or thumb-printed by each caretaker.

## Results

### Sample characteristics

There were substantial baseline differences between the three countries (Tables 1 and 2). Considering factors such as years of schooling, electricity and socio-economic status, participants in Burkina Faso were the poorest and those in South Africa were least poor: 85% of women in Burkina Faso had no education, compared to 7% in Uganda and < 1% in South Africa. The same country differences were also seen for maternal body size. Further, in Uganda, participants were somewhat poorer in the intervention arm than in the control arm. The effect of the intervention on absolute change in EBF prevalence varied substantially between countries. The largest effect was seen in Burkina Faso and Uganda. Around 12 weeks, the following differences in EBF prevalence according to a 24-hour recall between the participants in the intervention and the control groups were observed in the three countries: Burkina Faso: 79% versus 35%; Uganda: 82% versus 44%; and South Africa: 10% versus 6% [18].

**Table 1:** Baseline characteristics of participants in the intervention arm and the control arm in each of the 3 countries, presents continuous data presented

	**Intervensjon**		**n**	**Control**
	**n**	**Mean (SD)**	**n**	**Mean (SD)**
**Maternal age in years**				
Burkina Faso	292	25.8 (6.5)	318	25.8 (6.6)
Uganda	393	25.8 (6.8)	363	25.6 (6.5)
South Africa	533	24.4 (6.3)	485	24.2 (6.1)
**Maternal education in years**				
Burkina Faso	377	0.8 (1.9)	379	0.7 (1.9)
Uganda	391	6.1 (3.1)	365	6.5 (3.5)
South Africa	535	10.1 (2.3)	485	10.0 (2.3)
**Maternal BMI**				
Burkina Faso	247	21.0 (2.0	214	21.9 (2.4)
Uganda	343	22.2 (2.9)	312	22.2 (2.7)
South Africa	432	26.8 (6.2)	398	26.4 (5.7)
Birth weight, kg*				
Burkina Faso	7	3.4 (0.8)	6	3.2 (0.8)
Uganda	127	3.3 (0.6)	142	3.4 (0.7)
South Africa	495	3.2 (0.5)	446	3.1 (0.5)

*The data are reported from health cards if measurements were taken by health staff and recordings available and illustrate very limited use of health facilities in Burkina Faso and also low use in Uganda. The PROMISE EBF team had no influence on the quality of the birth weight measurements.

**Table 2:** Baseline characteristics of participants in the intervention arm and the control arm in each of the 3 countries, categorical data presented

	**Intervention**	**Control**
	**n (%)**	**n (%)**
**Two upper socio-economic quintiles**		
Burkina Faso	157/392 (40.1)	158/401 (39.4)
Uganda	132/396 (33.3)	172/369 (46.6)
South Africa	221/535 (41.3)	213/485 (43.9)
**Having access to toilet facility**		
Burkina Faso	3/168 (1.8)	3/232 (1.3)
Uganda	255/339 (75.2)	269/328 (82.0)
South Africa	433/515 (84.1)	389/456 (85.3)
**Having electricity**		
Burkina Faso	18/389 (4.6)	5/402 (1.2)
Uganda	53/391 (13.6)	70/361 (19.4)
South Africa	409/535 (76.5)	445/485 (91.8)
**Having access to non-surface water**		
Burkina Faso	271/388 (69.9)	239/400 (59.8)
Uganda	256/392 (65.3)	266/363 (73.3)
South Africa	414/535 (77.4)	379/484 (78.3)
**Attended antenatal clinic**		
Burkina Faso	284/389 (73.0)	285/401 (71.1)
Uganda	272/376 (72.3)	274/352 (77.8)
South Africa	527/532 (99.1)	470/481 (97.7)
**Delivery in health facility**		
Burkina Faso	143/372 (38.4)	128/370 (34.6)
Uganda	173/381 (45.4)	205/351 (58.4)
South Africa	486/514 (94.6)	423/461 (91.8)
**Female infant**		
Burkina Faso	188/392 (48.0)	199/401 (49.6)
Uganda	194/394 (49.2)	181/368 (49.2)
South Africa	256/531 (48.2)	256/479 (53.4)
**Having siblings**		
Burkina Faso	327/391 (83.6)	337/402 (83.8)
Uganda	311/392 (79.3)	281/366 (76.8)
South Africa	274/535 (51.2)	238/485 (49.1)

### Ponderal growth

A statistically significant adjusted difference between the arms was observed for WLZ in Burkina Faso at 12 weeks (Table 3). This difference was also present at 24 weeks when the mean WLZ (95% confidence intervals) was −0.74 (−0.87 to −0.60) in the intervention arm compared to −0.53 (−0.67 to −0.40) in the control arm: an adjusted difference of −0.20 (−0.39 to −0.01). In Uganda the corresponding estimates were 0.03 (−0.12 to 0.17) in the intervention arm compared to 0.28 (0.13 to 0.43) in the control arm: an adjusted difference of −0.23 (−0.43 to −0.03). In South Africa, on the other hand, the adjusted mean WLZ was 0.23 (0.03 to 0.43) higher in the intervention than in the control arm at 24 weeks.

**Table 3:** Weight-for-length (WLZ), weight-for-age (WAZ) and length-for-age (LAZ) least squares means (Mean) z-scores at 3, 6, 12 and 24 weeks from longitudinal data analysis

	**Intervention**	**Control**	**Crude**^**a**^	**Adjusted**^**b**^
	**Mean (95% CI)**	**Mean (95% CI)**	**Difference (95% CI)**	**Difference (95% CI)**
**WLZ**				
**3 weeks**				
Burkina Faso	−0.76 (−0.90;-0.62)	−0.80 (−0.94;-0.66)	0.04 (−0.16;0.23)	0.04 (−0.16;0.24)
Uganda	−0.08 (−0.23;-0.06)	0.01 (−0.14;0.16)	−0.09 (−0.29;0.11)	−0.07 (−0.27;0.13)
South Africa	0.54 (0.38;0.70)	0.58 (0.41;0.75)	−0.04 (−0.27;0.19)	−0.02 (−0.25;0.21)
**6 weeks**				
Burkina Faso	−0.64 (−0.77;-0.52)	−0.56 (−0.68;-0.44)	−0.09 (−0.26;0.09)	−0.08 (−0.26;0.09)
Uganda	0.05 (−0.07;0.17)	0.13 (<0.01;0.26)	−0.08 (−0.25;0.08)	−0.06 (−0.23;0.10)
South Africa	0.61 (0.48;0.74)	0.56 (0.42;0.70)	0.05 (−0.13;0.23)	0.07 (−0.12;0.25)
**12 weeks**				
Burkina Faso	−0.52 (−0.65;-0.39)	−0.28 (−0.40;-0.15)	−0.24^*^ (−0.42;-0.07)	−0.24^*^ (−0.42;-0.06)
Uganda	0.20 (0.07;0.33)	0.29 (0.16;0.43)	−0.09 (−0.27;0.08)	−0.07 (−0.25;0.11)
South Africa	0.69 (0.56;0.82)	0.52 (0.37;0.66)	0.17 (−0.01;0.36)	0.19^*^ (0.01;0.38)
**24 weeks**				
Burkina Faso	−0.74 (−0.87;-0.60)	−0.53 (−0.67;-0.40)	−0.21^*^ (−0.40;-0.01)	−0.20^*^ (−0.39;-0.01)
Uganda	0.03 (−0.12;0.17)	0.28 (0.13;0.43)	−0.25^*^ (−0.45;-0.06)	−0.23^*^ (−0.43;-0.03)
South Africa	0.62 (0.48;0.76)	0.41 (0.26;0.56)	0.21^*^ (0.01;0.41)	0.23^*^ (0.03;0.43)
**LAZ**				
**3 weeks**				
Burkina Faso	−0.65 (−0.79;-0.52)	−0.63 (−0.76;-0.49)	−0.03 (−0.22;0.16)	−0.03 (−0.21;0.15)
Uganda	−0.32 (−0.45;-0.18)	−0.21 (−0.35;-0.06)	−0.11 (−0.30;0.08)	−0.09 (−0.28;0.11)
South Africa	−0.87 (−1.03;-0.71)	−0.83 (−1.00;-0.65)	−0.04 (−0.27;0.19)	−0.06 (−0.29;0.18)
**6 weeks**				
Burkina Faso	−0.58 (−0.71;-0.46)	−0.62 (−0.75;-0.50)	0.04 (−0.13;0.22)	0.04 (−0.13;0.21)
Uganda	−0.35 (−0.48;-0.22)	−0.21 (−0.34;-0.07)	−0.14 (−0.32;0.03)	−0.11 (−0.30;0.07)
South Africa	−0.71 (−0.85;-0.57)	−0.68 (−0.83;-0.53)	−0.03 (−0.23;0.17)	−0.04 (−0.25;0.16)
**12 weeks**				
Burkina Faso	−0.54 (−0.67;-0.42)	−0.66 (−0.78;-0.53)	0.11 (−0.07;0.29)	0.12 (−0.06;0.30)
Uganda	−0.45 (−0.58;-0.31)	−0.26 (−0.41;-0.12)	−0.18 (−0.37;0.002)	−0.15 (−0.34;0.04)
South Africa	−0.46 (−0.61;-0.31)	−0.43 (−0.58;-0.27)	−0.03 (−0.24;0.18)	−0.04 (−0.25;0.18)
**24 weeks**				
Burkina Faso	−0.87 (−1.00;-0.74)	−0.85 (−0.98;-0.72)	−0.02 (−0.20;0.17)	−0.02 (−0.20;0.16)
Uganda	−0.78 (−0.93;-0.63)	−0.61 (−0.77;-0.46)	−0.17 (−0.37;0.04)	−0.13 (−0.33;0.06)
South Africa	−0.23 (−0.38;-0.08)	−0.08 (−0.23;0.07)	−0.15 (−0.36;0.06)	−0.13 (−0.34;0.08)
**WAZ**				
**3 weeks**				
Burkina Faso	−0.94 (−1.07;-0.81)	−0.94 (−1.07;-0.82)	0.0 (−0.18;0.18)	0.0 (−0.18;0.18)
Uganda	−0.26 (−0.39;-0.13)	−0.09 (−0.23;0.04)	−0.17 (−0.34;0.01)	−0.14 (−0.33;0.05)
South Africa	−0.33 (−0.46;-0.19)	−0.26 (−0.41;-0.12)	−0.06 (−0.26;0.14)	−0.05 (−0.25;0.15)
**6 weeks**				
Burkina Faso	−0.90 (−1.02;-0.78)	−0.87 (−0.99;-0.76)	−0.03 (−0.20;0.15)	−0.03 (−0.20;0.15)
Uganda	−0.26 (−0.39;-0.14)	−0.08 (−0.21;0.04)	−0.18 (−0.34;0.02)	−0.15 (−0.32;0.03)
South Africa	−0.19 (−0.32;-0.06)	−0.19 (−0.33;0.05)	0.0 (−0.18;0.19)	0.01 (−0.17;0.20)
**12 weeks**				
Burkina Faso	−0.89 (−1.01;-0.76)	−0.82 (−0.94;-0.69)	−0.07 (−0.25;0.10)	−0.07 (−0.25;0.10)
Uganda	−0.31 (−0.44;-0.18)	−0.09 (−0.23;0.04)	−0.21^*^ (−0.39;-0.04)	−0.18 (−0.36;0.01)
South Africa	0.03 (−0.10;0.16)	−0.06 (−0.20;0.08)	0.09 (−0.10;0.28)	0.10 (−0.09;0.29)
**24 weeks**				
Burkina Faso	−1.15 (−1.29;-1.02)	−1.01 (−1.14;-0.87)	−0.15 (−0.34;0.04)	−0.15 (−0.34;0.05)
Uganda	−0.55 (−0.70;-0.41)	−0.25 (−0.41;-0.09)	−0.30^* ^(-0.51;-0.10)	−0.26^* ^(-0.44;-0.08)
South Africa	0.22 (0.07;0.36)	0.14 (−0.02;0.30)	0.08 (−0.14;0.29)	0.09 (−0.13 to 0.30)

^a^Controlled for clusters and repeated measurements from same individual.

^b^Controlled for as ^a^and adjusted for socio-economic status.

^*^p < 0.05.

At the 12 week assessment, wasting was almost twice as common in the intervention compared to in the control arms in both Burkina Faso and Uganda (Table 4). Adjusted prevalence ratios were 1.86 (1.09 to 3.19) in Burkina Faso and 1.98 (0.99 to 3.93) in Uganda. At the 24 week assessment the corresponding estimates were 1.40 (CI 0.84 to 2.32) in Burkina Faso, and 2.36 (1.11 to 5.00) in Uganda. There were no statistically significant differences in wasting prevalence in South Africa at 12 and 24 weeks.

**Table 4:** Wasting (WLZ < −2), underweight (WAZ < −2) and stunting (LAZ < −2) comparing the intervention and control arm and prevalence ratios (PR)

	**Intervention**	**Control**	**Crude**^**a**^	**Adjusted**^**b**^
	**n (%)**	**n (%)**	**PR (95% CI)**	**PR (95% CI)**
**Wasting**				
**3 week**				
Burkina Faso	48/330 (14.55)	59/335 (17.61)	0.77 (0.44-1.32)	0.76 (0.45-1.29)
Uganda	15/280 (5.36)	14/265 (5.28)	0.90 (0.44-1.84)	0.96 (0.47-1.95)
South Africa	18/298 (6.04)	2/247 (0.81)	7.66^‡ ^(2.30-25.49)	7.54^‡^ (2.17-26.25)
**6 week**				
Burkina Faso	35/348 (10.06)	37/353 (10.48)	0.98 (0.53-1.78)	0.96 (0.53-1.71)
Uganda	12/331 (3.63)	8/293 (2.73)	1.36 (0.62-2.99)	1.25 (0.59-2.67)
South Africa	6/387 (1.55)	7/344 (2.03)	0.80 (0.28-2.23)	0.79 (0.25-2.45)
**12 week**				
Burkina Faso	39/357 (10.92)	22/365 (6.03)	1.89^*^ (1.08-3.29)	1.86^*^ (1.09-3.19)
Uganda	22/361 (6.09)	10/316 (3.16)	2.04^*^ (1.04-4.01)	1.98 (0.99-3.93)
South Africa	11/434 (2.53)	12/394 (3.05)	0.93 (0.46-1.87)	0.84 (0.42-1.69)
**24 week**				
Burkina Faso	47/353 (13.3)	37/361 (10.2)	1.41 (0.82-2.45)	1.40 (0.84-2.32)
Uganda	26/344 (7.56)	10/316 (3.16)	2.39^*^ (1.09-5.24)	2.36^*^ (1.11-5.00)
South Africa	8/351 (2.28)	6/302 (1.99)	1.28 (0.34-4.75)	1.12 (0.30-4.11)
**Stunting**				
**3 week**				
Burkina Faso	39/333 (11.71)	42/340 (12.35)	0.90 (0.58-1.38)	0.89 (0.57-1.41)
Uganda	33/283 (11.66)	18/265 (6.79)	1.75 (0.86-3.56)	1.67 (0.85-3.31)
South Africa	45/301 (14.95)	49/248 (19.76)	0.77 (0.58-1.03)	0.78 (0.59-1.04)
**6 week**				
Burkina Faso	37/349 (10.60)	40/356 (11.24)	0.91 (0.60-1.39)	0.90 (0.60-1.38)
Uganda	36/334 (10.78)	20/296 (6.76)	1.55 (0.89-2.71)	1.45 (0.87-2.40)
South Africa	61/387 (15.76)	52/346 (15.03)	1.17 (0.84-1.62)	1.19 (0.86-1.65)
**12 week**				
Burkina Faso	46/358 (12.85)	51/366 (13.93)	0.81 (0.55-1.20)	0.81 (0.55-1.20)
Uganda	49/364 (13.46)	29/316 (9.18)	1.57 (0.86-2.86)	1.46 (0.80-2.67)
South Africa	62/435 (14.25)	59/395 (14.94)	0.97 (0.68-1.39)	0.96 (0.66-1.41)
**24 week**				
Burkina Faso	59/353 (16.71)	57/361 (15.79)	1.08 (0.73-1.61)	1.07 (0.73-1.57)
Uganda	71/344 (20.64)	48/316 (15.19)	1.39 (0.93-2.08)	1.28 (0.86-1.90)
South Africa	42/352 (11.93)	33/303 (10.89)	1.11 (0.70-1.75)	1.07 (0.70-1.65)
**Underweight**				
**3 week**				
Burkina Faso	53/341 (15.54)	48/341 (14.08)	1.04 (0.65-1.65)	1.03 (0.64-1.64)
Uganda	22/285 (7.72)	14/268 (5.22)	1.50 (0.70-3.20)	1.30 (0.64-2.61)
South Africa	22/310 (7.10)	18/262 (6.87)	1.08 (0.62-1.89)	1.08 (0.62-1.90)
**6 week**				
Burkina Faso	59/351 (16.81)	58/358 (16.20)	1.11 (0.71-1.75)	1.10 (0.71-1.71)
Uganda	23/333 (6.91)	12/293 (4.10)	1.52 (0.70-3.30)	1.44 (0.67-3.08)
South Africa	26/395 (6.58)	29/351 (8.26)	0.81 (0.46-1.40)	0.84 (0.47-1.47)
**12 week**				
Burkina Faso	58/357 (16.25)	49/365 (13.42)	1.09 (0.73-1.61)	1.09 (0.74-1.59)
Uganda	37/361 (10.25)	17/316 (5.38)	2.04 (0.98-2.24)	1.80 (0.92-3.52)
South Africa	26/435 (5.98)	32/396 (8.08)	0.79 (0.49-1.29)	0.77 (0.48-1.24)
**24 week**				
Burkina Faso	75/354 (21.19)	64/361 (17.73)	1.23 (0.88-1.72)	1.23 (0.88-1.71)
Uganda	56/345 (16.23)	32/317 (10.09)	1.67 (0.86-3.22)	1.52 (0.81-2.88)
South Africa	18/351 (5.13)	15/306 (4.90)	1.21 (0.59-2.51)	1.18 (0.58-2.38)

^a^Controlled for cluster, site and ipw (inverse probability weights).

^b^Controlled for cluster, site, ipw and socio-economic status.

^‡^p ≤ 0,001, ^*^p < 0.05.

### Linear growth

The differences in mean LAZ between the intervention and control arms were small (<0.15) and not statistically significant (Table 3). No statistically significant differences were seen in the prevalence estimates for stunting between arms in any of the countries at any of the visits (Table 4).

### Weight-for-age z-scores, underweight and weight change

In Uganda, the mean WAZ was lower in the intervention arm than in the control arm: an adjusted difference of −0.26 (−0.44 to −0.08) at 24 weeks (Table 3). The adjusted difference in mean weight in grams (95% CI) at 24 weeks in Uganda was -211 g (−332 to −9) while in Burkina Faso it was -97 g (−215 to 21). There were no statistically significant differences observed in the prevalence estimates for underweight between arms in any of the countries at any of the visits (Table 4).

### Standard deviations

The standard deviations for WLZ ranged from 1.2 to 1.3 and that for LAZ from 1.2 to 1.5 over the scheduled visits in the 3 countries (Additional file 1).

## Discussion

This paper presents growth patterns including ponderal and linear growth and weight information of children up to six months of age who participated in a community-based trial assessing promotion of EBF by peer counsellors in Burkina Faso, Uganda and South Africa. Although the reported EBF prevalence doubled at 12 weeks in the intervention arms in all three countries, the absolute increase was large in Burkina Faso and Uganda (44 and 38 percentage points, respectively) and small in South Africa (4 percentage points) [18]. The child growth patterns varied in the three countries, with South Africa having the highest z-scores on average and Burkina Faso having the lowest. Ponderal growth tended to be slightly lower in Burkina Faso and Uganda in the intervention arms compared to the control arm even if the absolute weight differences were quite small at 24 weeks, around 100 g and statistically not significant in Burkina Faso, and around 200 g in Uganda at six months. Wasting was also more common at 24 weeks of age in Uganda. No significant differences were found for LAZ-scores between children in the intervention and control arms.

Compared to individual randomisation, cluster randomisation is more vulnerable to suboptimal randomisation as fewer units are randomised, and there is a chance that particular characteristics may be clustered [36]. However, the intra-cluster coefficients for the main outcomes were small [18]. Residual confounding from factors which we were not able to assess is still possible; for instance, as many of the births took place at home, gestational age and birth weight were available only for a small proportion of the mother-infant pairs in Burkina Faso and Uganda. Some would argue that our anthropometric measurements at around 3 weeks could act as a proxy for the corresponding baseline characteristics. However, there were only small differences in WLZ at 3 weeks of age and adjusting for 3 week weight (data not shown) did not substantially alter our effect estimates. It is known that societal, maternal and individual factors are related to growth outcomes [35,37,38], and some of these may have been unmeasured and unevenly distributed between the study arms. However, adjusting for socio-economic status, which is likely to capture some of these characteristics, only minimally altered our effect measures. Missing data were most common at 3 weeks because many mothers left their homes and stayed with their relatives for some time after giving birth. An *inverse-probability weighted method* yielded similar results compared to an *available-subject analysis* (data not shown) indicating no noteworthy bias from missing data [39].

There were major country differences with regard to the effect of the intervention on EBF prevalence [18], and as reported in this paper, socio-economy, maternal education and BMI as well as in infant growth patterns. There were also country differences with respect to perinatal mortality [40-42]. Thus, we find it most appropriate to present the results by country although pooling the data would have increased our statistical precision. As the absolute difference in EBF prevalence between the arms in South Africa was very small, it is difficult to attribute any differences in growth patterns to the EBF promotion. The country specific contextual challenges explaining this low uptake of EBF has been described [43] as well as poor breastfeeding practices [44]. The peer support for families to obtain a social welfare grant provided in the control clusters is also unlikely to have mitigated child growth.

The infants in the Multicentre Growth Reference Study (MGRS) study [45], which yielded the reference against which our infants’ growth was assessed, had non-smoking mothers from middle class or ‘affluent’ environments supportive of healthy growth [46]. In that study, 75% were exclusively or predominantly breastfed for 4 months and nearly 70% breastfed for a year. In our study, children from both Burkina Faso and Uganda were at 12 weeks of age exclusively breastfed to the same or even to a higher extent. The children in the PROMISE EBF trial were believed to represent the general population from their respective study areas and were not purposely selected from well-to-do families. In both arms of the PROMISE EBF trial in Burkina Faso and Uganda, we observed a gradual and substantial shift of the distributions towards poorer linear growth with increasing age, with a mean LAZ between −0.6 and −0.9 at 24 weeks. This growth pattern is described also in other studies in sub-Saharan Africa [37].

In Burkina Faso and Uganda, the prevalence of wasting was slightly higher in the intervention arms as compared to the control arms at 12 and 24 weeks. This finding informs the debate launched by Kramer and colleagues who also found an average weight reduction associated with EBF, and could not rule out an increased risk of undernutrition [3,5]. This could indicate that our intervention was inappropriate for the most vulnerable children. Even if the difference in mean WLZ of the children in the intervention and the control arms in Burkina Faso and Uganda was similar at 24 weeks, the distribution of the WLZ of the children in the intervention arm in Burkina Faso was skewed towards lower values, away from the WHO growth standard mean, while in Uganda, the mean WLZ among children in the intervention arm was closer to the WHO WLZ mean. A shift towards lower WLZ might benefit populations in which obesity is common [16], but in Uganda, where most communities are challenged by widespread undernutrition, the long term health consequences of a possible impairment in ponderal growth could be a concern.

The one week training course equipped the peer counsellors with basic information on promoting and supporting EBF, thus increasing EBF prevalences substantially in Burkina Faso and Uganda [24]. However, the peer counsellors had relatively low educational level and did not have other training in health care. Further, they often operated in environments with limited infrastructure with respect to water and sanitation, and where the public health system was not optimal, accessible and equitable, contributing to wasting, stunting and underweight [47]. Further research is needed to address the role and qualifications of peer counsellors for EBF to provide them with support to deliver safe interventions [30].

Our research group has described, particularly in Uganda, how poverty and food insecurity is an important challenge to proper child feeding [48,49]. This is also a problem in Burkina Faso where the anthropometric status of children seems to be even worse [50]. It is also known that formula feeding is mostly unaffordable, unacceptable and unfeasible in both Burkina Faso and Uganda [49], so access to industry formula cannot explain any of our findings. Neither can increased diarrhoea morbidity [18]. Even if the intervention has been described as acceptable by mothers in Uganda [51], it is not fully understood how the intervention altered feeding behaviour. However, a recent quantitative description from the Promise EBF trial on changes in feeding categories at the different time points [52] informs us that there are shifts in all directions with time between the different feeding categories. It is important that future studies address how an infant feeding intervention might change both maternal and infant diets and behaviour.

Breastfeeding of infants up to 6 months of age was nearly universal in both Burkina Faso and Uganda [53]. This intervention might mainly have shifted infants from being predominantly or partially breastfed to being exclusively breastfed [52]. However, it is important to assess how the observed changes have impacted on morbidity and mortality. Some studies highlight that a shift from predominant to exclusive breastfeeding up to six months does not add any health benefits [9]. A trial in Guinea-Bissau, despite challenges with high losses to follow-up, saw higher EBF prevalence in the intervention arm, but did not find a beneficial impact on diarrhoea prevalence, mortality or infant weight [54]. In fact, in the subset (40%) of children that were weighed close to 6 months of age, the median weight in the intervention group was slightly lower than in the control group. Likewise, our PROMISE EBF trial found no effect on diarrhoea morbidity [18]. Assessment of mortality did not indicate clear differences but were not powered for comparing the trial arms [40,41]. Although our trial protocol did not calculate sample size for child growth we think the sample size was sufficient for this intention-to-treat analysis on growth patterns. This is supported by a post hoc power calculation indicating high power, the narrow confidence intervals (Additional file 1) and high follow-up rates (Figure 1).

Follow-up studies are needed to assess long-term growth and health patterns for these children in order to balance potential negative and positive effects of EBF promotion is these settings. This will inform WHO’s global nutrition target towards 2025 aiming at reducing malnutrition [55].

## Conclusion

There were small differences in growth patterns between the study arms in the three countries. In both Burkina Faso and Uganda, children in the intervention clusters had slightly lower ponderal growth at 24 weeks of age. Beneficial health effects or absence of negative impact on morbidity, mortality and infant growth reported in some EBF promotion trials in Asia have yet to be demonstrated in Africa. There is a need to better understand 1) the reasons for this discrepancy, 2) how to optimize infant feeding support in Sub-Saharan Africa, where predominant breastfeeding and undernutrition is common, and 3) how to ensure that breastfeeding promotion can effectively be combined with appropriate referral of vulnerable children for nutritional support and disease management.

## Abbreviations

CI: Confidence intervals; EBF: Exclusive breastfeeding; LAZ: Length-for-age z-scores; LSM: Least Squares Means; MGRS: Multicentre Growth Reference Study; WAZ: Weight-for-age z-scores; WHO: World Health Organization; WLZ: Weight-for-length z-scores.

## Competing interests

The authors declare that they have no competing interests.

## Authors’ contributions

Among the authors, IMSE, DJ, AHD, TD, JN, DS, NM, JKT, ECE, PVdP, CK, HS and TT designed the study and the intervention. IMSE, DJ, LTF, VN, TT and HS planned and wrote the paper. IMSE and LTF handled, cleaned and analysed the data. SS and CL were study statisticians. DS contributed to manuscript design and content. AHD, VN and TD had particular responsibility for study implementation and data quality and management in Burkina Faso, Uganda and South Africa, respectively. HW contributed to data management and VR contributed to analytical content. ECE, NM, JKT, PvP, CK and DJ were country principal investigators and planned the study design, administered implementation and worked on analytic content. TT was the central PI. All authors read and contributed towards the final manuscript.

## Pre-publication history

The pre-publication history for this paper can be accessed here:

http://www.biomedcentral.com/1471-2458/14/633/prepub

## Supplementary Material

Additional file 1 Information on data management, including cleaning and missing data; inverse-probability weighting; standard deviations and post- hoc power calculation. Click here for file
