# Supplementary material for: Growth effects of exclusive breastfeeding promotion by peer counsellors in sub-Saharan Africa: the cluster-randomised PROMISE EBF trial
Source: BMC Public Health. 2014 Jun 21;14:633. doi: 10.1186/1471-2458-14-633 (PMC4082276; doi:10.1186/1471-2458-14-633)
Supplement: Additional file 1 — Information on data management, including cleaning and missing data; inverse-probability weighting; standard deviations and post- hoc power calculation. [file 1471-2458-14-633-S1.doc]

# Additional file 1. Information on data management, including cleaning and missing data; inverse-probability weighting; standard deviations and post- hoc power calculation

Recruitment, inclusion and exclusion criteriaIn Uganda, eligible women were identified by recruiters in the study areas and thereafter approached for data collection. In Burkina Faso and South Africa, a random sample of the eligible mothers in the intervention clusters were approached for data collection as the number of women receiving the intervention in the clusters exceeded the sample size requirements for the primary outcomes of the EBF study. Those intending to breastfeed and planning to stay in the selected cluster in the forthcoming year were included. Women were recruited as early as possible in the last trimester and no later than one week after delivery. Exclusion criteria were severe psychological illness which could interfere with consent or trial collaboration, having given birth more than one week before the first data collection contact and severe malformations that could interfere with breastfeeding.

Data cleaning and handling of missing data.

Data cleaning

Anthropometric data was assessed and cleaned in two stages:

During stage 1, the absolute differences for length and weight between measurements were checked for outliers. A maximum negative length difference between two measurements was set to 1.5 cm from the scheduled 6 week interview and to the proceeding interviews and to 2.0 cm from the scheduled 3 to 6 weeks interviews. This measurement error was allowed accounting for difficulties measuring babies as young as 1.5 week in their home settings and knowing that some were premature and small for gestational age.

The second stage of cleaning was based on the attained z-scores yielded from the WHO Child Growth Standards. Measurements were regarded as implausible based on the individual values if:

1) WAZ <-6 or > 5;

2) LAZ <-6 or > 6;

3) WLZ <-5 or > 5 or

4) WLZ > 3 and LAZ <-3.

Extreme changes in LAZ- and WLZ-scores of more than 2.5 or 3 from one interview to the next was also regarded as implausible.

All these extreme values were assessed individually. Unless no justification for the criteria above were found (e.g. a note indicating marasmus or a consistent small child), the corresponding measurements were set to missing.

Within each country the difference in missing between arms was less than 5% per scheduled visit except for LAZ at 12 weeks in Uganda with a difference of 6%. A summary of the distribution of missing data across arms per scheduled visit for WLZ, LAZ and WAZ was assessed. To assess whether missing anthropometric information resulted in a selection bias, baseline characteristics were compared between all participants and a slightly smaller group with no missing values for LAZ at 12 weeks. There was some uneven distribution of certain socio-economic characteristics suggesting that missing might not have been completely random. This latter finding justified the use of an inverse-probability weighted method. A short description of this technique follows below:

Inverse-probability weighted methods

Missing information was more common at the 3 week interview (13–38%) than later. At 12 weeks, missing information ranged from 8–17%, and at 24 weeks it ranged from 9–22%, with the least missing data in Burkina Faso and most in South Africa.

As a *complete-subject analysis* only includes those with existing data for certain models, another more pragmatic term used in our manuscript is *available-subject-analysis*, which is only recommended when values are *missing at random*. The alternative options include using an imputation technique or an inverse-probability weighted method. As imputation could introduce substantial uncertainty into the models as we would need to impute weight, length and age, the latter option was chosen. The cases were given a weight according to their likelihood of participation in the study. This was estimated based on having or not having other measurements at other time points, arm allocation and socio-demographic characteristics involving site, socio-economic status, having access to non-surface water, having access to electricity, mother’s education, mother’s age, parity, gender of infant, marital status, being weighted at birth, place of delivery, feeding plans before birth and feeding status at 12 or 24 weeks. Country and interview specific probability indices for missing, *pi,* were constructed using the *probit* command in Stata (probability regression models). The inverse-probability weighted variables *ipw* were equal to *1/pi* and incorporated as *pweight* into the models giving more weight to those with valid data that inverse characteristics of those with the highest probability of having missing data. Both *available-subject analysis,* only including those with existing data for certain models, and models incorporating *inverse-probability weighted methods* were calculated for comparisons. Results from the latter assessment are reported when the data are analysed in the *wide* format. When the *long* format was used, weighting was not incorporated as the likelihood of having data points was higher. The choice of using *inverse-probability weighted methods* was partly based on the general discussion by Rothman, Greenland and, Lash: Modern epidemiology p. 219

Overview of mean WLZ and LAZ in the respective countries with standard deviations (SD) per scheduled visits.

The standard deviations for WLZ ranged from 1.2 to 1.3 and that for LAZ from 1.2 to 1.5 over the scheduled visits in the 3 countries which indicates that length and weight were measured with adequate reproducibility.

|  |  | **WLZ** | | | **LAZ** | | |
| --- | --- | --- | --- | --- | --- | --- | --- |
| **Week** | **Country** | **N** | **Mean** | **SD** | **N** | **Mean** | **SD** |
| 3 (1.5- <4.5) | Burkina Faso | 670 | -0.83 | 1.2 | 678 | -0.65 | 1.2 |
|  | Uganda | 568 | -0.09 | 1.2 | 571 | -0.38 | 1.2 |
|  | South Africa | 587 | -0.49 | 1.3 | 592 | -0.82 | 1.4 |
| 6 (4.5-<9) | Burkina Faso | 703 | -0.53 | 1.3 | 707 | -0.63 | 1.2 |
|  | Uganda | 642 | 0.15 | 1.2 | 648 | -0.34 | 1.2 |
|  | South Africa | 803 | 0.74 | 1.3 | 805 | -0.34 | 1.2 |
| 12 (9-<18) | Burkina Faso | 723 | -0.43 | 1.2 | 725 | -0.65 | 1.2 |
|  | Uganda | 683 | 0.17 | 1.3 | 685 | -0.42 | 1.3 |
|  | South Africa | 853 | 0.54 | 1.3 | 855 | -0.42 | 1.4 |
| 24 (18-28) | Burkina Faso | 721 | -0.63 | 1.2 | 721 | -0.85 | 1.2 |
|  | Uganda | 675 | 0.08 | 1.3 | 675 | -0.80 | 1.4 |
|  | South Africa | 803 | 0.55 | 1.3 | 805 | -0.17 | 1.5 |

Post-hoc power calculation

A post-hoc calculation of power for our analysis is given below, generally this was very high, except for LAZ in Burkina Faso where the intervention and control arms were almost similar. Calculations were made according to [www.statisticalsolutions.net](http://www.statisticalsolutions.net/) and the following given: A 2-tailed test, alpha of 0.05, the differences in mean, the varying number of measured children at 24 weeks (Table 1) and the corresponding standard deviation given above gave the following power:

WLZ: Burkina Faso: 100%, Uganda: 100%, and South Africa: 100%

LAZ: Burkina Faso: 7%, Uganda: 89%, and South Africa: 83%.
